# Interventions to improve social network in older people with sensory impairment: a systematic review

**DOI:** 10.1007/s40520-024-02695-w

**Published:** 2024-02-12

**Authors:** Li Kuang, Hanyu Hu, Halina Dai, Huiying Ma, Yuling Jia, Yu Sheng

**Affiliations:** 1 School of Nursing, Chinese Academy of Medical Sciences & Peking Union Medical College, No.33 of Badachu Road, Shijingshan District, Beijing, 100144 People’s Republic of China; 2 Peking Union Medical College Hospital, Chinese Academy of Medical Sciences & Peking Union Medical College, Beijing, 100730 China

**Keywords:** Social network, Older people, Sensory impairment, Systematic review

## Abstract

**Background:**

Sensory impairment significantly reduces speech discrimination and perception ability, presenting a challenge to effective communication. It can lead to social withdrawal and a reduced social network which can lead to cognitive impairment, seriously affecting the quality of life of older people. However, it is unclear which intervention components are effective to improving social network in older people with sensory impairment.

**Objective:**

The aim of this systematic review was to summarize interventions designed to improve social network in older people with sensory impairment.

**Methods:**

We searched seven databases from inception to December 1, 2023. Eligible studies included randomized clinical trials (RCT) and quasi-experimental studies of interventions for older people with sensory impairment aimed at improving social networks. Two reviewers searched databases, extracted data, and assessed the quality of the included studies independently.

**Results:**

Nine studies including five RCTs and four quasi-experimental studies were selected, enrolling 721 older people with sensory impairment. Methodological quality of the studies was modest. Eight studies demonstrated a positive impact of the interventions used in older people with sensory impairment. The detailed effective intervention components may include communication strategies, resources for older people and their significant others, exercise or tech-back of communication, sensory device fitting, and use and maintenance of sensory devices.

**Conclusion:**

There are few interventions available for improving social network in older people with sensory impairment. Most interventions mainly focus on communication education and care, and sensory device fitting and education. To improve the social network in older people with sensory impairment, it is necessary to develop more effective, multidisciplinary collaborative effective interventions and conduct more high-quality original studies.

**Supplementary Information:**

The online version contains supplementary material available at 10.1007/s40520-024-02695-w.

## Introduction

Population aging has resulted in a notable epidemiological transition marked by an increase in the prevalence of chronic disease [1]. A considerable proportion of older people experience age-related hearing and/or vision impairment [2], with a notable proportion experiencing dual sensory impairment (DSI), which is the combination of hearing and vision impairment [3]. According to World Health Organization (WHO) criteria, hearing impairment refers to hearing impairment more than 25 decibels in ears with better hearing, and vision impairment refers to vision below 0.3 logMAR (e.g., 20/40 or 6/12) [4]. Any degree of vision and hearing impairment, regardless of severity, age, or order of onset, is defined as DSI [3, 5, 6]. WHO estimated that more than 1.5 billion people worldwide are affected by hearing impairment, with 430 million experiencing moderate to severe hearing impairment [7]. Vision impairment or blindness affect at least 2.2 billion people worldwide [8]. However, this number is expected to increase in the coming years due to a growing and aging population.

Evidence suggests that sensory impairment has a significant negative influence on older people’s health and may result in undesirable health outcomes [9, 10], which are associated with high healthcare utilization and mortality [11]. Older people suffer not only physical deterioration but also mental harm because of old age and sensory impairment [12]. It is crucial to note that sensory impairment experiences are inevitably linked to the interpersonal context of the individual and can profoundly affect and be affected by close family members, particularly spouses or romantic partners [13–15].

Sensory impairment restricts older people’s ability to communicate and limits their participation in daily activities. More specifically, older people with sensory impairment may miss words and meanings as well as be unable to read body language, facial expressions, and lip movements during talks, which can cause close relationships to suffer from fatigue, frustration, stress, or resentment in close relationship [16–18]. Furthermore, sensory impairment can lower a person’s quality of life and involvement in society, cause them to feel isolated and embarrassed, and disrupt their relationships with family and friends which may increase risk of depression and cognitive impairment [19]. However, most studies neglect to examine the social network between older people with sensory impairment, especially when there are additional disorders present. Therefore, it is essential to implement interventions for the social network in older people with sensory impairment.

Various interventions have been studied to improve the social network in older people with sensory impairment, including hearing aids, lenses, and participation in social activities. However, the effectiveness of these interventions and their specific components remains unclear due to variations in sample size, intervention content, and research findings. No systematic review focuses on how to improve the social network in older people with sensory impairment. Therefore, a rigorous systematic review is necessary to explore the interventions to improve the social network in older people with sensory impairment.

This systematic review aims to summarize the studies related to improving the social network in older people with sensory impairment. The review will include randomized clinical trials and quasi-experimental studies, and will analyze the intervention components, the intervention delivers, the support network involved and the role, and efficacy of the interventions to provide an evidence-based reference for clinical research.

## Methods

The systematic review was registered with the International Prospective Register of Systematic Reviews (PROSPERO registration number: CRD42023420696). It was conducted in accordance with the Preferred Reporting Items for Systematic Review and Meta-analyses (PRISMA) guidelines [20].

### Search strategy

The search strategy was developed collaboratively with the support of the second author. The English database Cochrane Library, MEDLINE, Web of Science, EMBASE and Chinese database China National Knowledge Infrastructure (CNKI), Chinese Biomedical Journal (CBM), and Wanfang database were searched from inception to 1 December 2023 using medical subject headings (MeSH) terms, text word searches, and Boolean calculation searches (see supplemental material Table S1). The search strategy included two key concepts: sensory impairment and social network, which were customized for each database.

### Eligibility criteria

Eligible studies must have met the inclusion criteria:

Types of studies: All of the full-text, randomized controlled trials (RCTs) or quasi-experimental studies that were included. The limited languages were Chinese and English.

(2) Population: Adult patients (age ≥ 60 years) with sensory impairment, including hearing impairment only, vision impairment only, or dual sensory impairment (hearing impairment and vision impairment).

(3) Intervention: Interventions for older people with sensory impairment aimed to improve social networks with different types of content, e.g., health education, hearing aids, etc.

(4) Outcomes: Studies used social networks as (one of) the primary outcome(s). If there was no clear difference between the primary and secondary outcomes, we included studies in which one or more of the outcomes were social network.

### Data extraction and synthesis

Two independent researchers extracted the data. Data included: (1) basic information of the included studies, including the first author and publication date, the number of participants, age, type of sensory impairment, reasons for missing, etc.; (2) intervention characteristics of the included studies in the control and experimental groups, including underlying framework, intervention content and delivers, support network involved and role, social network measures, etc.; (3) components of the included interventions. Detailed basic characteristics and summary of the included literature are shown in Table 1, Table 2, and Table 3.

**Table 1:** Basic characteristics of included studies

Studies	Study design	Participant	Age of participants[mean ± SD(range)]	Group Pre/post	Reasons for missing
Kramer et al., 2005 (Netherlands) [28]	RCT	Older adults with hearing impairment	E:69.00 ± 7.70 C:71.00 ± 8.50	E:29/24 C:29/24	E: drop out(5) C: drop out(5)
Hickson et al., 2007 (Australia) [31]	RCT	Older people with hearing impairment	E:73.68 ± 8.62 C:74.10 ± 7.90	E:78/70 C:100/97	Not presented
Öberg et al., 2017 (Sweden) [30]	Quasi-experimental study	Older people with hearing impairment	73.90 ± 9.80	102/77	Never started the program(7); Not attended enough session and left the intervention (18)
Deal et al., 2017 (America) [23]	RCT	Older people with hearing impairment	70–84 (not detailed)	E:20/20 C:20/19	C:death(1)
Mamo et al., 2017 (America) [26]	Quasi-experimental study	Older dementia people with hearing impairment	76.90 ± 12.40	20/20	No missing
Choi et al., 2019 (America) [27]	Quasi-experimental study	Older people with hearing impairment	67.90 ± 8.10	22/15	Lose contact after screening (4); schedule conflict (3)
Leroi et al., 2020 (Ireland) [24]	Quasi-experimental study	Older dementia people with hearing or/and vision impairment	76.00(65.00,87.00)	19/19	No missing
Vreeken et al., 2020 (Netherlands) [25]	RCT	Older people with hearing and vision impairment	E:81.30 ± 9.90 C:81.90 ± 10.00	E:64/54 C:67/57	E:measurements incomplete(9); C:measurements incomplete(9)
Nieman et al., 2022 (America) [29]	RCT	Older people with hearing impairment	E:75.70 ± 7.00 C:77.70 ± 8.90	E:78/73 C:73/70	E: discontinued study (4); lost to follow-up (1) C: discontinued study (2); lost to follow-up (1)

*E* experimental group, *C* control group, *RCT* randomized controlled trial

**Table 2:** Intervention characteristics of included studies

Studies	Underlying framework	Control group	Intervention group	Intervention deliver(s)	Setting where intervention were delivered	Support network involved and role	Social network measures	Impact of intervention on social network
Kramer et al., 2005 (Netherlands) [28]	No presented	Hearing aid fitting	Home education program; hearing aid fitting; 1-2 weeks per film, 5–12 weeks (11 ± 3.7 weeks)	Self-administered	Home	Significant others; participate in intervention and practice the communication strategies together	HHDI-reaction of others	Compared with control group, the training group had significantly better interaction with significant others
Hickson et al., 2007 (Australia) [31]	Not presented	Social program + ACE program	ACE program 2 h/week over 5 weeks	Researchers	Homes or at a community center	Significant others (e.g., spouses, relatives); participate in intervention	The Quantified Denver Scale of Communicative Function	Compared with control group, the ACE group showed significant pre-to-post-improvements on the Self-Assessment of Communication
Öberg et al., 2017 (Sweden) [30]	Not presented	No control group	Swedish ACE program: 6–10 participants, 2 h sessions a week, five week	Audiologists and hearing therapists	Clinic	Significant others (e.g., spouses, relatives); participate in intervention	CSS	Individuals who attended with a significant other tended to use better communication strategies statistically. The qualitative results indicated that the program increased individuals’ ability to cope and restored their social identities
Deal et al., 2017 (America) [23]	Not presented	Successful aging intervention	Evidence-based best practices; four 1-h session over a period of 10-12 weeks	Study audiologists and a research nurse	Not presented	Communication partners or adults who communicate with participants on a daily or near (e.g., spouse)	Number of people and diversity	There is no significant change in social network of the hearing intervention group
Mamo et al., 2017 (America) [26]	Bandura’s Social Cognitive Theory	No control group	Memory-hear intervention; one 2-h session	Researchers	Outpatient setting	Significant others; attended the group session between the participant and the interventionist	Semi-structured interviews	Qualitative responses from caregivers described improved engagement for their loved ones, such as laughing more, telling more stories, asking more questions, and having more patience
Choi et al., 2019 (America) [27]	Bandura’s Social Cognitive Theory	No control group	K-Hear program(Hear program for Korea Americans); a one-time session	Researchers	Community	Communicate partners; received the education about hearing device and communication	Focus group after intervention with participants and their communication partners	The participants discussed impact of the program on themselves, family members and their community and reported significant hearing benefit in many situations. And communication partners noted their own change in attitudes when dealing with the residual frustrating situation due to their partner’s hearing loss
Leroi et al., 2020 (Ireland) [24]	‘COM-B Behavior Change model	No control group	Sensory intervention; 16 visits, over 12 weeks	Audiologists and/or optometrists and researchers	Home or clinic	Study partners coresident or in regular contact (at least twice a week);participate in intervention	RSS	There was a modest improvement (in absolute terms) post-intervention in social network
Vreeken et al., 2020 (Netherlands) [25]	Not presented	Care as usual	DSL protocol; 3–5 weekly session, 3–5 weeks	Occupational therapists	Home	Communication partners; participated in 3–5 session training	HHDI-reaction of others	There was significantly improvements in social network
Nieman et al., 2022 (America) [29]	Bandura’s Social Cognitive Theory	Care as usual	HEAR program; 1 to 2 sessions over less than 2 h, 3 months	Community health worker	Community	Communicate partners; participated in intervention delivered by community health workers	SNI	Compared with control group, the participants in the intervention group reported no significant improvements in the social network

*ACE program* active communication education program, *DSL* dual sensory loss, *CSS* the communication strategies scale, *RSS* relationship satisfaction scale, *HHDI-reaction of others* the 10-item Hearing Handicap and Disability Inventory Reaction of Others’ subscale, *SNI* social network index

**Table 3:** Components of included interventions

Components												
	Communication education and care intervention						Sensory device fitting and education intervention					
	Necessity of communication	Setting communication goals	Communication strategies	Resources for older people and their significant others	Exercise or tech-back of communication	Other social care	Sensory function screening	Sensory impairment education	Sensory device needs assessment	Sensory device fitting	Use and maintenance of sensory device	Supplementary aids
Interventions with whole social network content												
Hickson et [31] al, 2007	√		√									
Öberg et al., 2017 [30]			√		√							
Interventions with partly social network content												
Kramer et al., 2005 [28]			√	√	√					√	√	√
Mamo et al., 2017 [26]		√	√		√			√			√	
Deal et al., 2017 [23]			√	√					√	√		
Choi et al., 2019 [27]	√	√	√	√			√	√	√	√		
Leroi et al., 2020 [24]			√		√	√	√			√	√	√
Vreeken et al., 2020 [25]			√							√		√
Nieman et al., 2022 [29]			√		√		√			√		

### Quality assessment

The same researchers independently assessed the methodological quality of the included studies using the Joanna Briggs Institute critical appraisal checklists for RCTs and quasi-experimental studies [21]. The RoB2.0 tool is designed with five domains to assess the bias of RCTs from different aspects, including the randomization process, deviations from intended interventions, missing outcome data, measurement of the outcome, and selection of the reported result [22]. Researchers completed a series of questions that assessed the risk of bias in each of these five domains for each study, and the RoB2.0 tool proposes a risk of bias score for each domain as either ‘low’, ‘some concerns’, or ‘high’ based on the algorithm. Quasi-experimental studies were assessed from nine domains, including causal relationships of research variables, baseline levels, intervention measures, controls, measurement of outcome indicators, follow-up, and analysis, which were answered with ‘yes’, ‘no’, ‘unclear’, and ‘not applicable’. If there was disagreement between two researchers, the third researcher (HD) was consulted.

## Results

### Search outcomes

**Fig. 1:**
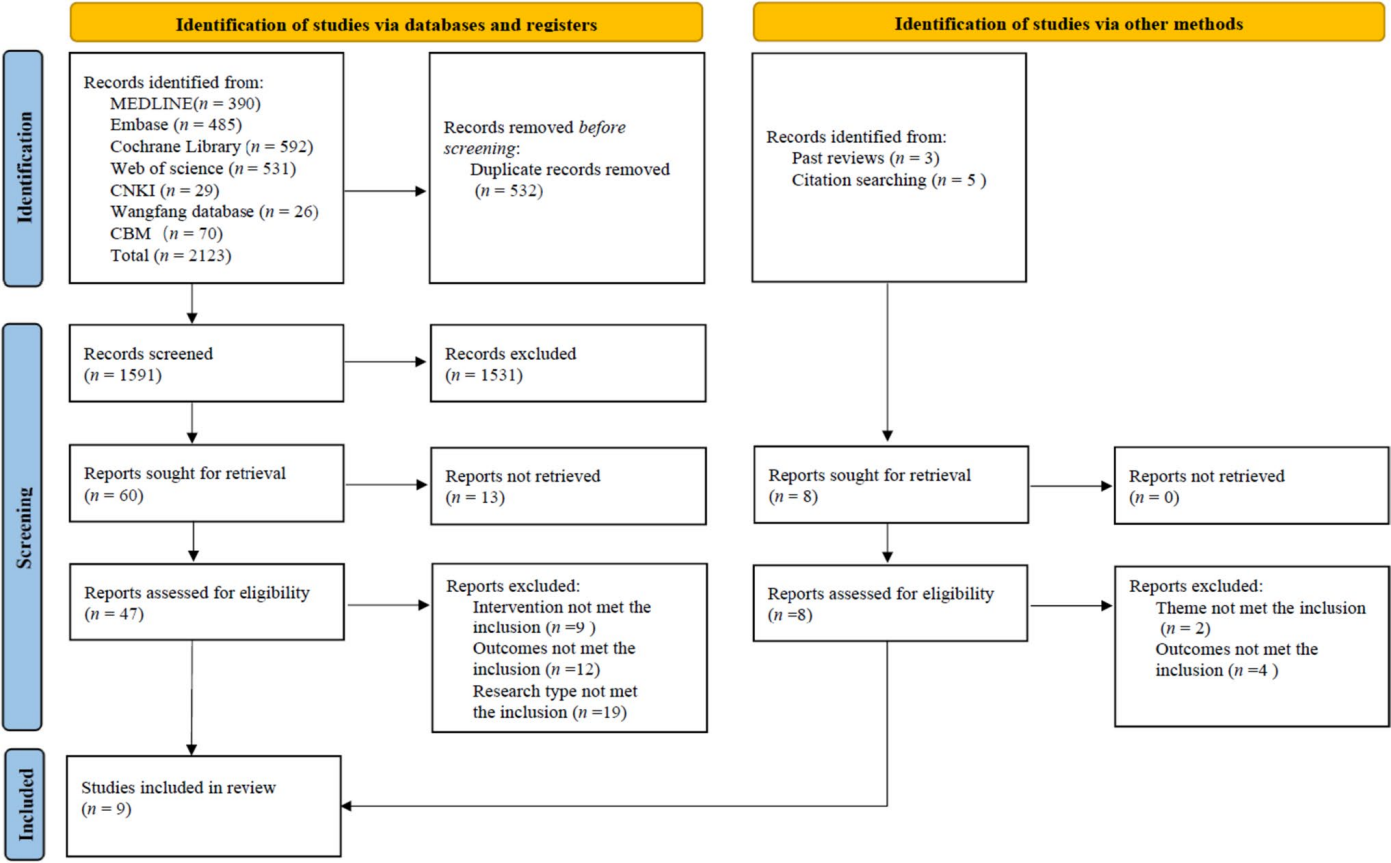
PRISMA flow diagram

A total of 2,123 studies were initially collected, including 1,998 English studies and 125 Chinese studies. After removing duplicates (*n* = 532) and screening for titles, abstracts and full texts, nine English studies finally were included. The process and results of literature research are shown in Fig. 1.

### Characteristic of included studies

The characteristics of the included studies are shown in Table 1. Of the included studies, five studies were RCTs, and four were quasi-experimental studies. Seven hundred twenty-one participants were included in the study, with sample sizes ranging from 19 to 178. The average age of included study also varied between studies, ranging from 67.9 to 81.9 years. Some studies only provided the age range without specifying the average age [23, 24], but the most common age range of participants was 65–82 years. The participants of seven studies were older people with hearing impairment, one study [24] included participants with hearing impairment and/or vision impairment, and another study [25] included participants with dual sensory impairment. Only in two studies [24, 26], participants were older people with dementia, and other studies included older people with normal cognitive function. Eight studies [23–30] reported the missing of participants, but one study [31] did not provide an explanation for missing.

### Interventions characteristics of all studies included

Almost all included articles described different interventions (see Table 2). Of the nine studies, four studies [24, 26, 27, 29] reported the underlying framework they used for the intervention, including Bandura’s Social Cognitive Theory and the COM-B Behavior Change model. Most studies were delivered in the home or community with education or sensory device fitting. The intervention delivers of included studies were varied, including researchers, occupational therapists, community health workers or self-administered, etc. All of the studies involved elderly individuals and their significant others, such as a spouse, family member, or friend, to company the participants to accept the interventions together. Although all the included studies assessed the improvements in social networks, the assessment tools used to measure social networks were different. As well as using different scales to measure social networks, some of the included studies also used the number and variety of people, interviews or focus groups to assess social networks [23, 26, 27]. Some studies showed that both family members and patients had good feedback on the improvement of social networks, who believed that significant others could be more patient in understanding the sensory difficulties of participants and learn how to improve communication through intervention resulting in better communication between them [26, 27, 30].

The specific intervention measures were varied and included the Active Communication Education Program (ACE program), HEAR program, the Dual Sensory Loss Protocol (DSL protocol), and Sensory Intervention. The duration of the intervention varied from 5 to 12 weeks. The frequency of intervention varied from 15 min to 2 h per time. The majority of the selected studies were conducted in America (*n* = 4) [23, 26, 27, 29] and the Netherlands (*n* = 2) [25, 28], of which three studies discussed the different national versions of HEAR program including the selection of an over-the-counter personal sound amplification product fitting and orientation to the device, and education about hearing impairment and communication strategies [26, 27, 29]. Two RCTs [30, 31] conducted an ACE program that consisted of a series of modules on everyday communication activities such as communication needs analysis, understanding conversations in background noise, communicating around the house, understanding people who do not speak clearly, etc. One study [24] conducted sensory intervention that included not only fitting and advice for sensory devices but also included additional components such as communication training, support for social inclusion, etc. One study conducted DSL protocol in participants’ homes weekly, including optimal use of hearing aids and adaptations to the living environment and use of effective communication strategies to cope with dual sensory loss [25]. Some studies [24, 25, 29–31] used home visits to deliver the intervention making it more convenient to deliver the discussion or training.

The interventions of all studies were divided into two categories based on the focus of intervention content. The interventions of two studies [30, 31] were all social network related, and the interventions of seven studies [23–29] were partially social network related. The components presented were: communication education and care intervention, sensory device fitting, and education intervention. A detailed description of the intervention is given in Table 3, which illustrates the main intervention components included in each study and the components of content included in each separate intervention.

#### Communication education and care intervention

All included studies had clear communication intervention content between participants and their significant others. The categories of communication education and care intervention presented were: necessity of communication, setting communication goals, communication strategies, resources for older people and their significant others, exercise or tech-back of communication, and other social care. Most of these studies reported specific communication strategies about how to communicate in real-world settings to raise awareness of communication problems between the patient and significant others and addresses adequate coping. Common communication strategies included understanding the hearing impairment, communicating in background noise, shortening the distance to the communication partner and reducing glare, using clarification or repetition of requests, and asking the communication partner to speak slowly or to articulate well, etc. In one study [26], significant others were asked to explain the communication strategies they used in everyday life during a tech-back session. The two studies [30, 31] that solely used interventions with social networks showed that older people with sensory impairment tended to use better community strategies than before or in the control group. Öberg et al. [30] found that the ability of participants to cope had been increased, and their social identities had been restored according to the qualitative findings. Kramer et al. [28] used five videotapes and an instruction booklet with increasing difficulty levels as a teaching tool and communication strategies and questions and issues for discussion in each chapter. It was a self-administered intervention that differed from other programs. Öberg et al. [30] conducted a Swedish version of the ACE program, which combined the features of the study by Hickson et al. [31] who initially implemented the ACE program. Three studies [26, 27, 29] using the same Hear program, which emphasized the importance of amplification device fitting and orientation, also added less communication content to improve the effect of sensory device fitting.

#### Sensory device fitting and education intervention

Of the included studies, seven studies [23–29] combined communication intervention with sensory device fitting and education intervention. The categories of sensory device fitting and education intervention presented were: sensory function screening, sensory impairment education, the sensory device needs assessment, sensory device fitting, and use and maintenance of the sensory device and supplementary aids. One study [26] only reported education related to sensory impairment without sensory device fitting content. Six studies [23–25, 27–29] reported the specific sensory device and education intervention, including sensory impairment screening, sensory device fitting, and sensory impairment education. Choi et al. [27] reported a 30-min screening for the ear to provide a proper sensory device for older people, which included basic ear examination with an otoscope, audiometric screening with an automated screening protocol in a quiet room, key clinical history of ear and hearing that may require additional evaluation and review of the limitations of the hearing screening and personal sound amplifier products used in the study (versus professionally fitted hearing aids). The included sensory education was divided into sensory impairment education and instruction on how to use and maintain an over-the-counter amplification device. Leroi et al. [24] found that it was necessary to provide the participants with supplementary aids to improve sensory function in the home environment, such as glasses straps, hearing aid clips, special lighting, and ambient noise management.

Of the seven studies [23–29], only one RCT by Deal et al. [23] showed that the social network of the hearing intervention group had no significant changes from before or from the control group. However, the study by Deal et al. [23] was a pilot study and it prepared the ground for more investigation. Choi et al. [27] conducted a quasi-experimental study which showed that communication partners in a focus group noted their own change in attitude when dealing with the residual frustration of their partner’s hearing impairment.

### Risk of bias assessment

**Fig. 2:**
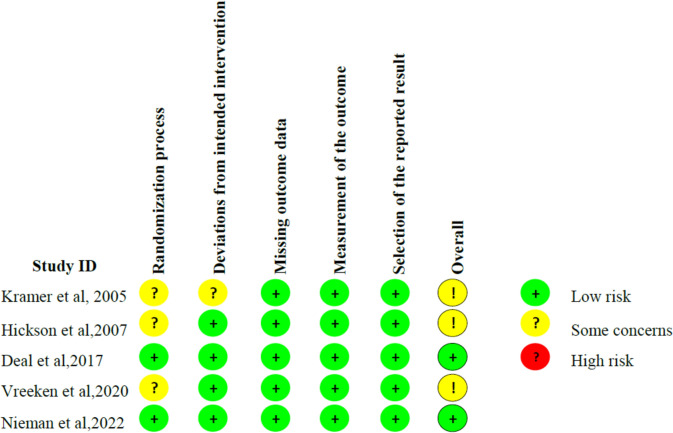
Details of each dimension of the risk of bias tool

**Fig. 3:**
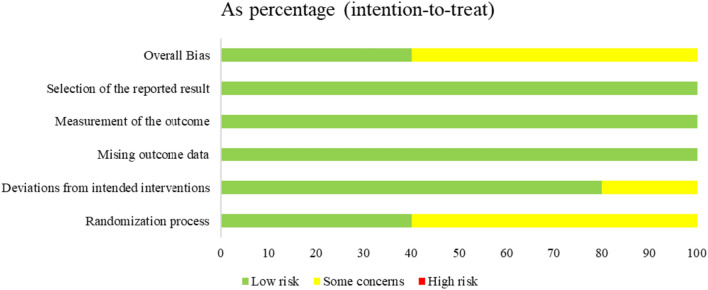
Risk of bias in the included studies

The risk of bias in the included studies was graded as moderate overall. Of the five studies [23, 25, 28, 29, 31], two studies [23, 29] (40%) were identified as ‘low risk’ using the RoB2.0 tool, and three studies [25, 28, 31] (60%) were classified as ‘some concerns’ (see Fig. 2, Fig. 3 and supplemental material Table S2). Three studies [25, 28, 31] only stated that participants were randomly grouped, but the specific process was not specified. All four quasi-experimental studies [24, 26, 27, 30] did not meet all quality criteria because they were single-arm intervention studies without a control group (see supplemental material Table S3).

## Discussion

We conducted this comprehensive systematic review of interventions to improve the social networks of older people with sensory impairment in the community. The results are of great importance because they can be used to further shape the care of older people with sensory impairment provided by multidisciplinary teams in evidence-based medicine.

The review identified nine studies including five RCTs and four quasi-experimental studies. Eight studies [24–31] demonstrated positive effects on the social networks in older people with sensory impairment. We identified effective intervention components by categorizing the included intervention content and dividing the content into two main aspects: communication education and care intervention, sensory device fitting and education intervention. The intervention components of communication education and care intervention used in the included studies involved the necessity of communication, setting communication goals, communication strategies, resources for older people and their significant others, exercise or tech-back of communication, and other social care. The intervention components of sensory device fitting and education intervention used in the included studies included: sensory function screening, sensory impairment education, the sensory device needs assessment, sensory device fitting, and use and maintenance of the sensory devices and supplementary aids.

However, some intervention components may not directly improve the social network, despite their frequent presence in the intervention content. Instead, they may improve the social network indirectly through other components. Therefore, it is necessary to further analyze the intervention components included in effective interventions to identify the true components that play a role. Comparing the inclusion of intervention components in effective versus non-effective interventions can help determine whether these components effectively improve the social network. Effective intervention components may include communication strategies, resources for older people and their significant others, exercise or tech-back of communication, sensory device fitting, and use and maintenance of sensory devices. These components are more likely to be included in effective studies. Studies have shown that sensory device fitting is the most common treatment option for older people with sensory impairment [32]. Sensory devices, especially hearing aids, has been found to improve social networks by increasing positive feelings such as happiness and reducing negative emotions such as frustration and fatigue [33].

A community-based survey in developed countries and regions found that around 10–20% of older adults with hearing impairment use hearing aids [34]. The limitation of this method is that many adults who experience hearing and communication difficulties in their daily lives, and those who are unable or unwilling to continue installing hearing aids at specific times, do not receive any intervention services [35]. People with sensory impairment were often isolated from friends and family due to reduced communication skills and increased communication difficulties, leading them to further restricted social networks and potentially exacerbating other diseases such as depression and dementia [36]. Previous studies have shown that older people with sensory impairment experienced more communication disruptions, many of which remain unresolved in many instances [37]. Therefore, to optimize communication effectiveness in this population, it is strongly recommended to develop a communication training plan for older people with sensory impairment and their communication partners to optimize. Our systematic review found that all nine studies included interventions in communication strategies, which were crucial for examining the social networks of older people with sensory impairment. To enhance the improvement of social network, the majority of the included studies in this systematic review combined communication education and care intervention with the sensory device fitting and education intervention. Better sensory knowledge is associated with greater psycho-social well-being, and strengthening communication strategy skills can empower sensory device users to practice the same skills in real life [38–40].

All studies indicated that having a significant other to participate in the intervention together was crucial. It is commonly recognized that sensory impairment, including hearing impairment, vision impairment, or dual sensory impairment, presents a significant challenge to maintaining intimate relationships due to its negative impact on couples’ functioning and their ability to communicate effectively [41, 42]. More specifically, barriers to daily communication between couples can increase the risk of emotional and sexual disconnection, leading to a decrease in perceived relationship satisfaction and endangering both members’ ability to adapt and maintain supportive relationships [15, 43–45]. It has been shown that behavioral change and the implementation of successful intrapersonal, interpersonal, and dyadic coping strategies can help to minimize the adverse consequences of sensory loss. Lazzarotto et al. [46] identified several strategies that spouses of older people with sensory impairment tend to use, which can be categorized into four main types: avoidance, positive thinking, problem-solving, and social seeking. On the dyadic level, it is essential for significant others to use communication strategies to overcome everyday communication difficulties such as repetition, attention seeking, and repositioning in conversation. Therefore, it is necessary for significant others to learn and practice these communication strategies.

Some studies were conducted in participants’ homes, which may be more conducive to creating a scene of daily life and better experiencing the effectiveness of the intervention to improve the social network in older people with sensory impairment. It is worth noting that one study [28] used a self-administered intervention approach, which differed from other studies. The intervention provided information to participants and their significant others, aiming to enhance self-management skills in sensory health for older people with sensory impairment. By empowering them to be as proactive and better adapt to the changes in their social network caused by sensory impairment [38], this intervention guided participants toward greater self-efficacy and it improved their ability to manage their sensory health.

## Limitations and future directions

However, there are several limitations of our study. First, we only included nine studies that were published in Chinese or English. We did not search grey literature, which might lead to missing some relevant studies. And we did not retrieve all full texts after initially identified relevant studies, because some studies are registered clinical trials with none of their results available and some studies were conference abstracts with no full texts. Despite exhaustive efforts including database searches and author contact, we were unable to obtain the full texts to include these studies in the analysis. In the future, we will continue to conduct searches and pay attention to updated relevant literature, to continuously expand the number of studies included and provide more evidence for the social network research of older people with sensory impairment. Second, there was significant variation among the included studies in terms of intervention methods, duration, frequency, and assessment tools for social networks. The high heterogeneity among the intervention groups prevented us from conducting a meta-analysis in this study. Due to the limited number of intervention studies available on the social network in older people with sensory impairment, the use of multiple intervention methods, and the predominance of studies in the pre-experimental stage, almost half of the included studies were quasi-experimental. Only five studies were RCTs and they had a small sample size. Therefore, the available evidence was insufficient. For future research, we recommend conducting more RCTs with larger sample sizes and establishing international collaborations to ensure more comprehensive and harmonized evaluations of the effectiveness of interventions aimed at improving the social network in older people with sensory impairment.

## Conclusion

The aim of this systematic review was to identify the potential interventions which could improve the social network in older people with sensory impairment through the identification and categorization of intervention content, the systematic review revealed that interventions targeting the improvement of the social network could be delivered by therapists and nurses. These intervention components mainly fell into two categories: communication education and care intervention, and sensory device fitting and education intervention. Effective intervention components may include communication strategies, resources for older people and their significant others, exercise or tech-back of communication, sensory device fitting, and use and maintenance of sensory devices. As the effectiveness of an intervention may be influenced by the interactions between its components, it is important to study these interactions. The suitable delivery methods for these intervention contents were information, group discussion, and training which could be delivered by nurses. To maximize positive effects of these interventions on the social network, further studies are required to explore the optimal delivery methods and long-term sustainability of the intervention effects on the social network. Furthermore, it is recommended to investigate whether such interventions are effective for all types of sensory impairment and whether medical staff can incorporate them into their daily practice. Therefore, large-scale and high-quality RCTs are still necessary to generate stronger evidence in the future.

### Supplementary Information

Below is the link to the electronic supplementary material.

Supplementary file1 (DOCX 68 KB)

## Data Availability

The data supporting the findings and conclusions of this systematic review are derived from information provided within the published studies included. A full list of the search terms and quality assessment of included studies can be found in the supplementary appendix. As no new data sets were created, none are available for further sharing.

## Declarations

### Research involves human and animals rights participants

This review reports no original findings or participant data that need ethics approval.
