# Supplementary material for: Interventions to improve social network in older people with sensory impairment: a systematic review
Source: Aging Clin Exp Res. 2024 Feb 12;36(1):34. doi: 10.1007/s40520-024-02695-w (PMC10861684; doi:10.1007/s40520-024-02695-w)
Supplement: Supplementary file 1 — Supplementary file1 (DOCX 68 KB) [file 40520_2024_2695_MOESM1_ESM.docx]

**Supplemental material**

**Table S1 Search strategies**

| **MEDLINE** | |
| --- | --- |
| #1 | "Aged"[MeSH Terms] |
| #2 | "Aged"[Title/Abstract] |
| #3 | "old people"[Title/Abstract] OR "elderly"[Title/Abstract] OR "geriatric"[Title/Abstract] OR "older"[Title/Abstract] |
| #4 | "Aged"[MeSH Terms] OR "Aged"[Title/Abstract] OR "old people"[Title/Abstract] OR "elderly"[Title/Abstract] OR "geriatric"[Title/Abstract] OR "older"[Title/Abstract] |
| #5 | \| "sensory loss"[Title/Abstract] OR "sensory impairment"[Title/Abstract] OR "sensory function"[Title/Abstract] OR "hearing impairment"[Title/Abstract] OR "hearing loss"[Title/Abstract] OR "vision loss"[Title/Abstract] OR "vision"[Title/Abstract] OR "hearing"[Title/Abstract] \| \| --- \| |
| #6 | \| #4 AND #5 \| \| --- \| |
| #7 | "social network"[Title/Abstract] OR "social support"[Title/Abstract] |
| #8 | #6 AND #7 |
| #9 | "family relations"[Title/Abstract] OR "family conflict"[Title/Abstract] OR "intergenerational relations"[Title/Abstract] OR "sibling relations"[Title/Abstract] OR "family"[Title/Abstract] OR "adult children"[Title/Abstract] OR "family role"[Title/Abstract] OR "family therapy"[Title/Abstract] |
| #10 | "husband"[Title/Abstract] OR "wife"[Title/Abstract] OR "relatives"[Title/Abstract] OR "family based"[Title/Abstract] |
| #11 | "couple"[Title/Abstract] OR "married"[Title/Abstract] OR "spous*"[Title/Abstract] OR "partner*"[Title/Abstract] OR "household"[Title/Abstract] OR "neighbor*"[Title/Abstract] |
| #12 | #9 OR #10 OR #11 |
| #13 | #6 AND #12 |
| #14 | #8 OR #13 |
| #15 | "trial*"[Title/Abstract] OR "clinical trial"[Title/Abstract] |
| #16 | #14 AND #15 |
| #17 | "intervention*"[Title/Abstract] |
| #18 | #14 AND #17 |
| #19 | #16 OR #18 |
| **Cochrane library** | |
| #1 | ("sensory loss" or "sensory impairment" or "sensory function" or "vision impairment" or "vision loss" or "vision" or "hearing loss" or "hearing impairment" or "hearing"):ti,ab,kw |
| #2 | ("old people" or "elderly" or "geriatric" or "old adult" or "older"):ti,ab,kw |
| #3 | MeSH descriptor: [Sensation] explode all trees |
| #4 | MeSH descriptor: [Aged] explode all trees |
| #5 | #1 or #3 |
| #6 | #2 or #4 |
| #7 | #5 and #6 |
| #8 | MeSH descriptor: [Social Networking] explode all trees |
| #9 | ("social network" or "social support"):ti,ab,kw |
| #10 | #8 or #9 |
| #11 | MeSH descriptor: [Family Relations] explode all trees |
| #12 | ("family conflict" or "intergenerational relations" or "sibling relations" or "family" or "adult childern" or "family role" or "family therapy"):ti,ab,kw |
| #13 | "husband" or "wife" or "relatives" or "family based" or "couple" or "married" or "spous*" or "partner*" or "household" or "neighbor*"):ti,ab,kw |
| #14 | #11 or #12 or #13 |
| #15 | #7 and #10 |
| #16 | #7 and #14 |
| #17 | #15 or #16 |
| #18 | ("trial*"):ti,ab,kw OR ("clinical trial"):pt |
| #19 | #17 and #18 |
| #20 | ("intervention*"):ti,ab,kw |
| #21 | #17 and #20 |
| #22 | #21 or #19 |
| **Web of science** | |
| #1 | TS=("old people") OR TS=("elderly") OR TS=("geriatric") OR TS=("old adult") OR TS=("older") OR TS=("aged") |
| #2 | TS=("sensory impairment") OR TS=("sensory loss") OR TS=("hearing loss") OR TS=("hearing impairment") OR TS=("vision loss") OR TS=("vision impairment") OR TS=("sensory function") OR TS=("hearing") OR TS=("vision") |
| #3 | TS=("social network") OR TS=("social support) |
| #4 | #1 AND #2 |
| #5 | #3 AND #4 |
| #6 | TS=("family conflict") OR TS=("family relations") OR TS=("family role") OR TS=("family therapy") OR TS=("intergenerational relation") OR TS=("sibling relation") OR TS=("family) OR TS=("adult childern") |
| #7 | #4 AND #6 |
| #8 | TS=("husband") OR TS=("married") OR TS=("spous*") OR TS=("partner*") OR TS=("household") OR TS=("neighbor*") |
| #9 | TS=("couple") OR TS=("wife") OR TS=("relatives") OR TS=("family based") |
| #10 | #4 AND #8 |
| #11 | #4 AND #9 |
| #12 | #5 OR #7 OR #10 OR #11 |
| #13 | TS=("clinical trial") OR TS=("trial") |
| #14 | #12 AND #13 |
| #15 | TS=("intervention*") |
| #16 | #12 AND #15 |
| #17 | #14 OR #16 |
| **EMBASE** | |
| #1 | ‘old people’:ab,ti OR ‘elderly’:ab,ti OR ‘geriatric’:ab,ti OR ‘old adult’:ab,ti OR ‘older’:ab,ti OR ‘aged’:ab,ti |
| #2 | ‘sensory impairment’:ab,ti OR ‘sensory loss’:ab,ti OR ‘sensory function’:ab,ti OR ‘vision impairment’:ab,ti OR ‘hearing impairment’:ab,ti OR ‘hearing loss’:ab,ti OR ‘vision loss’:ab,ti |
| #3 | #1 AND #2 |
| #4 | ‘social network’:ab,ti OR ‘social support’:ab,ti |
| #5 | #3 AND #4 |
| #6 | ‘family relations’:ab,ti OR ‘family conflict’:ab,ti OR ‘intergenerational relations’:ab,ti OR ‘sibling relations’:ab,ti OR ‘family’:ab,ti OR ‘adult childern’:ab,ti OR ‘family therapy’:ab,ti |
| #7 | #3 AND #6 |
| #8 | ‘husband’:ab,ti OR ‘wife’:ab,ti OR ‘relatives:ab,ti OR ‘family based’:ab,ti |
| #9 | ‘couple’:ab,ti OR ‘married’:ab,ti OR ‘spous*’:ab,ti OR ‘partner’:ab,ti OR ‘household’:ab,ti OR ‘neighbor*’:ab,ti |
| #10 | #3 AND #8 |
| #11 | #3 AND #9 |
| #12 | #5 OR #7 OR #10 OR #11 |
| #13 | ‘clinical trial’:ab,ti OR ‘trial’:ab,ti |
| #14 | #12 AND #13 |
| #15 | ‘intervention*’:ab,ti |
| #16 | #12 AND #15 |
| #17 | #14 OR #16 |
| **CNKI** | |
| #1 | SU %= '中老年人' OR SU %= '老人' OR SU %= '老年' OR SU %= '中年' |
| #2 | SU %= '感官障碍' OR SU %= '感觉障碍' OR SU %= '感官受损' OR SU %= '感觉障碍' OR SU %= '感觉受损' OR SU %= '听力障碍' OR SU %= '听力受损' OR SU %= '听觉障碍' OR SU %= '听力衰退' OR SU %= '视力障碍' OR SU %= '视力受损' OR SU %= '视力衰退' |
| #3 | SU %= '社会网络' OR SU %= '社会支持' OR SU %= '社会关系网络' OR SU %= '家人' OR SU %= '配偶' OR SU %= '夫妻' OR SU %= '子女' OR SU %= '儿女' OR SU %= '邻居' |
| #4 | FT = '干预 |
| #5 | #1 AND #2 AND #3 AND #4 |
| **Wanfang** | |
| #1 | 主题=‘老年人’ OR ‘老年’ OR ‘老人’ |
| #2 | 主题= ‘感官障碍’ OR ‘感觉障碍’ OR ‘感官受损’ OR ‘听力障碍’ OR ‘听力受损’ OR ‘视力障碍’ OR ‘视力受损’ OR ‘视力衰退’ OR ‘听力衰退’ |
| #3 | 主题= ‘社会网络’ OR ‘社会支持’ OR ‘社会关系网络’ OR ‘家人’ OR ‘朋友’ OR ‘配偶’ OR ‘夫妻’ OR ‘子女’ OR ‘邻居’ |
| #4 | 全部= ‘干预’ |
| #5 | #1 AND #2 AND #3 AND #4 |
| **CBMdisc** | |
| #1 | ‘老年人’[常用字段:智能] OR ‘老年’[常用字段:智能] OR ‘老人’[常用字段:智能] |
| #2 | ‘感官障碍’[常用字段:智能] OR ‘感觉障碍’[常用字段:智能] OR ‘感官受损’ [常用字段:智能] OR ‘感觉受损’[常用字段:智能] OR ‘听力受损’ [常用字段:智能] OR ‘听力障碍’[常用字段:智能] OR ‘视力受损’[常用字段:智能] OR ‘视力障碍’[常用字段:智能] |
| #3 | ‘社会网络’[常用字段:智能] OR ‘社会支持’ [常用字段:智能] OR ‘社会关系网络’[常用字段:智能] OR ‘家人’[常用字段:智能] OR ‘朋友’[常用字段:智能] OR ‘配偶’[常用字段:智能] OR ‘夫妻’[常用字段:智能] OR ‘子女’[常用字段:智能] OR ‘邻居’[常用字段:智能] |
| #4 | ‘干预’[常用字段:智能] |
| #5 | #1 AND #2 AND #3 AND #4 |

**Table S2 Details of critical appraisal of studies included in final systematic review according to Joanne Briggs Institute**

**Randomized Controlled Trails**

| Authors, Year, country | Domain 1 | | | Domain 2 | | | | | | | Domain 3 | | | | Domain 4 | | | | | Domain 5 | | |
| --- | --- | --- | --- | --- | --- | --- | --- | --- | --- | --- | --- | --- | --- | --- | --- | --- | --- | --- | --- | --- | --- | --- |
|  | Randomization process | | | Deviations from intended interventions | | | | | | | Missing outcome data | | | | Measurement of the outcome | | | | | Selection of the reported result | | |
|  | S  1.1 | S  1.2 | S  1.3 | S  2.1 | S  2.2 | S  2.3 | S  2.4 | S  2.5 | S  2.6 | S  2.7 | S  3.1 | S  3.2 | S  3.3 | S  3.4 | S  4.1 | S  4.2 | S  4.3 | S  4.4 | S  4.5 | S  5.1 | S  5.2 | S  5.3 |
| Kramer  et al,2005  (Netherlands) | Y | NI | N | NI | PY | NA | NA | Y | Y | NA | Y | NA | NA | NA | N | N | PN | NA | NA | Y | N | N |
| Hickson  et al,2007  (Australia) | Y | NI | N | N | N | NA | NA | NA | Y | NA | Y | NA | NA | NA | N | N | N | NA | NA | Y | N | N |
| Deal et al,2017  (America) | Y | Y | N | Y | Y | NA | N | NA | NA | NA | Y | NA | NA | NA | N | N | N | NA | NA | Y | N | N |
| Vreeken et al,2020  (Netherlands) | Y | N | N | Y | N | N | NA | NA | Y | NA | Y | NA | NA | NA | N | N | N | NA | NA | Y | N | N |
| Nieman,2022  (America) | Y | Y | N | N | N | NA | NA | NA | Y | NA | Y | NA | NA | NA | N | N | N | NA | NA | Y | N | N |

Note:

The items were based on the revised Cochrane risk of bias tool for randomized controlled trials (RoB 2.0). Specifically, the signaling questions involved are listed as follows:

S 1.1 = Signaling question 1.1: Was the allocation sequence random?

S 1.2 = Signaling question 1.2: Was the allocation sequence concealed until participants were enrolled and assigned to interventions?

S 1.3 = Signaling question 1.3: Did baseline differences between intervention groups suggest a problem with the randomization process?

S 2.1 = Signaling question 2.1: Were participants aware of their assigned intervention during the trial?

S 2.2 = Signaling question 2.2: Were carers and people delivering the interventions aware of participants’ assigned intervention during the trial?

S 2.3 = Signaling question 2.3: If Y/PY/NI to 2.1 or 2.2: Were there deviations from the intended intervention that arose because of the experimental context?

S 2.4= Signaling question 2.3: If Y/PY to 2.3: Were these deviations likely to have affected the outcome?

S 2.5= Signaling question 2.4: If Y/PY/NI to 2.4: Were these deviations from intended intervention balanced between groups?

S 2.6 = Signaling question 2.6: Was an appropriate analysis used to estimate the effect of assignment to intervention?

S 2.7 = Signaling question 2.6: If N/PN/NI to 2.6: Was there potential for a substantial impact (on the result) of the failure to analyse participants in the group to which they were randomized?

S 3.1 = Signaling question 3.1: Were data for this outcome available for all, or nearly all, participants randomized?

S 3.2 = Signaling question 3.2: If N/PN/NI to 3.1: Is there evidence that the result was not biased by missing outcome data?

S 3.3 = Signaling question 3.3: If N/PN to 3.2: Could missingness in the outcome depend on its true value?

S 3.4 = Signaling question 3.4: If Y/PY/NI to 3.3: Is it likely that missingness in the outcome depended on its true value?

S 4.1 = Signaling question 4.1: Was the method of measuring the outcome inappropriate?

S 4.2 = Signaling question 4.2: Could measurement or ascertainment of the outcome have differed between intervention groups?

S 4.3 = Signaling question 4.3: If N/PN/NI to 4.1 and 4.2: Were outcome assessors aware of the intervention received by study participants?

S 4.4 = Signaling question 4.4: If Y/PY/NI to 4.3: Could assessment of the outcome have been influenced by knowledge of intervention received?

S 4.5 = Signaling question 4.5: If Y/PY/NI to 4.4: Is it likely that assessment of the outcome was influenced by knowledge of intervention received?

S 5.1 = Signaling question 5.1: Were the data that produced this result analyzed in accordance with a pre-specified analysis plan that was finalized before unblinded outcome data were available for analysis?

S 5.2 = Signaling question 5.2: Is the numerical result being assessed likely to have been selected, on the basis of the results, from multiple eligible outcome mea-

surements (e.g. scales, definitions, time points) within the outcome domain?

S 5.3 = Signaling question 5.3: Is the numerical result being assessed likely to have been selected, on the basis of the results, from multiple eligible analyses of the data?

Abbreviations for assessment results:

(1) Y=Yes.

(2) PY=Probably yes.

(3) PN=Probably no.

(4) N=No.

(5) NI=No information.

(6) NA=Not applicable.

**Quasi-experimental studies**

| Studies | 1. Is it clear in the study what is the ‘cause’ and what is the ‘effect’ (i.e. there is no confusion about which variable comes first)? | 2.Were the participants included in any comparisons similar? | 3.Were the participants included in any comparisons receiving similar treatment/care, other than the exposure or intervention of interest? | 4.Was there a control group? | 5.Were there multiple measurements of the outcome both pre and post the intervention/exposure? | 6.Was follow up complete and if not, were differences between groups in terms of their follow up adequately described and analyzed? | 7.Were the outcomes of participants included in any comparisons measured in the same way? | 8.Were outcomes measured in a reliable way? | 9.Was appropriate statistical analysis used? |
| --- | --- | --- | --- | --- | --- | --- | --- | --- | --- |
| Mamo et al, 2017 | yes | yes | yes | no | yes | yes | yes | yes | yes |
| Oberg et al, 2017 | yes | yes | yes | no | yes | yes | yes | yes | yes |
| Choi et al, 2019 | yes | yes | yes | no | yes | yes | yes | yes | yes |
| Leroi et al, 2020 | yes | yes | yes | no | yes | yes | yes | yes | yes |

| **Section/topic** | **#** | **Checklist item** | **Reported on page #** |
| --- | --- | --- | --- |
| **TITLE** | | |  |
| Title | 1 | Identify the report as a systematic review, meta-analysis, or both. | 1 |
| **ABSTRACT** | | |  |
| Structured summary | 2 | Provide a structured summary including, as applicable: background; objectives; data sources; study eligibility criteria, participants, and interventions; study appraisal and synthesis methods; results; limitations; conclusions and implications of key findings; systematic review registration number. | 1-2 |
| **INTRODUCTION** | | |  |
| Rationale | 3 | Describe the rationale for the review in the context of what is already known. | 3 |
| Objectives | 4 | Provide an explicit statement of questions being addressed with reference to participants, interventions, comparisons, outcomes, and study design (PICOS). | 3-4 |
| **METHODS** | | |  |
| Protocol and registration | 5 | Indicate if a review protocol exists, if and where it can be accessed (e.g., Web address), and, if available, provide registration information including registration number. | 5 |
| Eligibility criteria | 6 | Specify study characteristics (e.g., PICOS, length of follow‐up) and report characteristics (e.g., years considered, language, publication status) used as criteria for eligibility, giving rationale. | 5-6 |
| Information sources | 7 | Describe all information sources (e.g., databases with dates of coverage, contact with study authors to identify additional studies) in the search and date last searched. | 5-7 |
| Search | 8 | Present full electronic search strategy for at least one database, including any limits used, such that it could be repeated. | 5-6 |
| Study selection | 9 | State the process for selecting studies (i.e., screening, eligibility, included in systematic review, and, if applicable, included in the meta‐analysis). | 6-7 |
| Data collection process | 10 | Describe method of data extraction from reports (e.g., piloted forms, independently, in duplicate) and any processes for obtaining and confirming data from investigators. | 6-7 |
| Data items | 11 | List and define all variables for which data were sought (e.g., PICOS, funding sources) and any assumptions and simplifications made. | 5-7 |
| Risk of bias in individual studies | 12 | Describe methods used for assessing risk of bias of individual studies (including specification of whether this was done at the study or outcome level), and how this information is to be used in any data synthesis. | 6-7 |
| Summary measures | 13 | State the principal summary measures (e.g., risk ratio, difference in means). | 6-7 |
| Synthesis of results | 14 | Describe the methods of handling data and combining results of studies, if done, including measures of consistency (e.g., I^2^ for each meta‐analysis.  ) | 6-7 |

Page 1 of 2

| **Section/topic** | **#** | **Checklist item** | **Reported on page #** |
| --- | --- | --- | --- |
| Risk of bias across studies | 15 | Specify any assessment of risk of bias that may affect the cumulative evidence (e.g., publication bias, selective reporting within studies). | 6-7 |
| Additional analyses | 16 | Describe methods of additional analyses (e.g., sensitivity or subgroup analyses, meta-regression), if done, indicating which were pre‐specified. | 6-7 |
| **RESULTS** | | |  |
| Study selection | 17 | Give numbers of studies screened, assessed for eligibility, and included in the review, with reasons for exclusions at each stage, ideally with a flow diagram. | 7 |
| Study characteristics | 18 | For each study, present characteristics for which data were extracted (e.g., study size, PICOS, follow-up period) and provide the citations. | 8-19 |
| Risk of bias within studies | 19 | Present data on risk of bias of each study and, if available, any outcome level assessment (see item 12). | 8-19 |
| Results of individual studies | 20 | For all outcomes considered (benefits or harms), present, for each study: (a) simple summary data for each intervention group (b) effect estimates and confidence intervals, ideally with a forest plot. | 8-19 |
| Synthesis of results | 21 | Present results of each meta-analysis done, including confidence intervals and measures of consistency. | 8-19 |
| Risk of bias across studies | 22 | Present results of any assessment of risk of bias across studies (see Item 15). | 8-19 |
| Additional analysis | 23 | Give results of additional analyses, if done (e.g., sensitivity or subgroup analyses, meta-regression [see Item 16]). | 8-19 |
| **DISCUSSION** | | |  |
| Summary of evidence | 24 | Summarize the main findings including the strength of evidence for each main outcome; consider their relevance to key groups (e.g., healthcare providers, users, and policy makers). | 20-23 |
| Limitations | 25 | Discuss limitations at study and outcome level (e.g., risk of bias), and at review-level (e.g., incomplete retrieval of identified research, reporting bias). | 23-24 |
| Conclusions | 26 | Provide a general interpretation of the results in the context of other evidence, and implications for future research. | 24-25 |
| **FUNDING** | | |  |
| Funding | 27 | Describe sources of funding for the systematic review and other support (e.g., supply of data); role of funders for the systematic review. | 25 |

*From:* Moher D, Liberati A, Tetzlaff J, Altman DG, The PRISMA Group (2009). Preferred Reporting Items for Systematic Reviews and Meta-Analyses: The PRISMA Statement. PLoS Med 6(6): e1000097. doi:10.1371/journal.pmed1000097

For more information, visit: **www.prisma‐statement.org**.

Page 2 of 2
